# RGD peptide-based lipids for targeted mRNA delivery and gene editing applications†

**DOI:** 10.1039/d2ra02771b

**Published:** 2022-09-07

**Authors:** Jingya Qin, Lulu Xue, Ningqiang Gong, Hanwen Zhang, Sarah J. Shepherd, Rebecca M. Haley, Kelsey L. Swingle, Michael J. Mitchell

**Affiliations:** Department of Bioengineering, University of Pennsylvania Philadelphia PA 19104 USA mjmitch@seas.upenn.edu; Abramson Cancer Center, Perelman School of Medicine, University of Pennsylvania Philadelphia Pennsylvania 19104 USA; Institute for Immunology, Perelman School of Medicine, University of Pennsylvania Philadelphia Pennsylvania 19104 USA; Cardiovascular Institute, Perelman School of Medicine, University of Pennsylvania Philadelphia Pennsylvania 19014 USA; Institute for Regenerative Medicine, Perelman School of Medicine, University of Pennsylvania Philadelphia Pennsylvania 19104 USA

## Abstract

mRNA therapeutics are promising platforms for protein replacement therapies and gene editing technologies. When delivered *via* non-viral gene delivery systems, such as lipid nanoparticles (LNPs), mRNA therapeutics are easy to produce and show low toxicity and immunogenicity. However, LNPs show limited delivery efficiency and tissue specificity in certain applications. To overcome this, we designed RGD peptide (Arg-Gly-Asp) based ionizable lipids, which can be formulated into LNPs for integrin binding on cells and targeted mRNA delivery. RGD-LNPs were formulated using microfluidic devices and screened *in vitro* for size, mRNA encapsulation efficiency, transfection efficiency, and cell viability. A lead candidate, 1A RGD-based hybrid LNP, showed effective mRNA encapsulation and transfection, and was selected for further testing, including the co-delivery of Cas9 mRNA and sgRNA for gene editing applications. *In vitro*, 1A RGD-based hybrid LNP outperformed a non-targeted control LNP and showed GFP knockout efficiencies up to 90%. Further, the improved cellular uptake was reversed in the presence of soluble RGD, supporting the hypothesis that this improved uptake is RGD-dependent. *In vivo*, 1A RGD-based hybrid LNPs showed comparable mRNA delivery to the liver and spleen, when compared to a non-targeted control, and had increased expression in the whole body. Overall, this RGD-based hybrid LNP system is a promising platform for targeted mRNA delivery, which may allow for mRNA-based protein replacement and gene editing in a more efficient and specific manner with reduced off-target effects.

## Abstract 2

We developed RGD peptide based ionizable lipids, which can be formulated into LNPs for integrin-dependent targeted mRNA delivery and gene editing applications.

## Graphical abstract

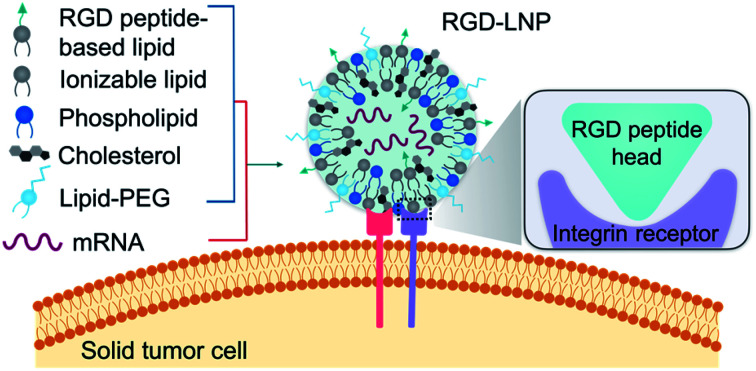

## Introduction

In recent years, messenger RNA (mRNA), a transient intermediate between genes and proteins, has emerged as a promising new approach for therapeutic applications including protein replacement therapies, vaccines, and gene editing applications.^1–3^ However, mRNA rapidly degrades and cannot easily cross the cell membrane due to its large size and negative charge.^4^ Therefore, the usage of mRNA therapeutics requires safe, effective, and stable delivery systems to protect from degradation and allow cellular uptake and functionality.^1,5–8^ In clinical trials, both viral and non-viral vectors are used for systemic delivery of mRNA.^9^ Viral vectors have high transfection efficiency and higher specificity for cell targeting, but have several intrinsic limitations including delayed cellular immune response, the possibility of carcinogenesis, and limited opportunity for repeated administration due to acute inflammatory response.^10–13^ In CRISPR-based genome editing specifically, non-viral systems for CRISPR–Cas9 delivery are preferred due to their improved biosafety profiles and the possibility for repeat dosages.^14,15^ However, low genome editing efficiency and shorter-lived gene expression using these non-viral systems remains a concern.^16^

Lipid nanoparticles (LNPs) represent a broad class of materials that are being investigated to deliver these mRNA therapeutics, including the co-delivery of mRNA Cas9 and single guide RNA (sgRNA).^17,18^ LNPs have been shown to provide potent, intracellular nucleic acid delivery across a variety of cell lines with minimal toxicity and immunogenicity, and are also easier to synthesize than viral vectors.^19–22^ LNPs are typically composed of four components: (i) ionizable amino lipids, which acquire charge at low pH, allowing for complexation with nucleic acids and eventual endosomal escape into the cytoplasm; (ii) phospholipids, which fortify the LNP bilayer structure and aid in endosomal escape; (iii) cholesterol, which enhances LNP stability and promotes membrane fusion; and (iv) a lipid-polyethylene glycol (PEG) conjugate, which inserts into the LNP bilayer and provides a PEG coating that reduces LNP aggregation and nonspecific endocytosis by immune cells.^23–26^ These four components are easily modified to influence the physicochemical properties of LNPs to affect their delivery and uptake into cells, and their variation may encompass 10^10^ or more distinct LNP formulations.^27,28^

**Fig. 1:**
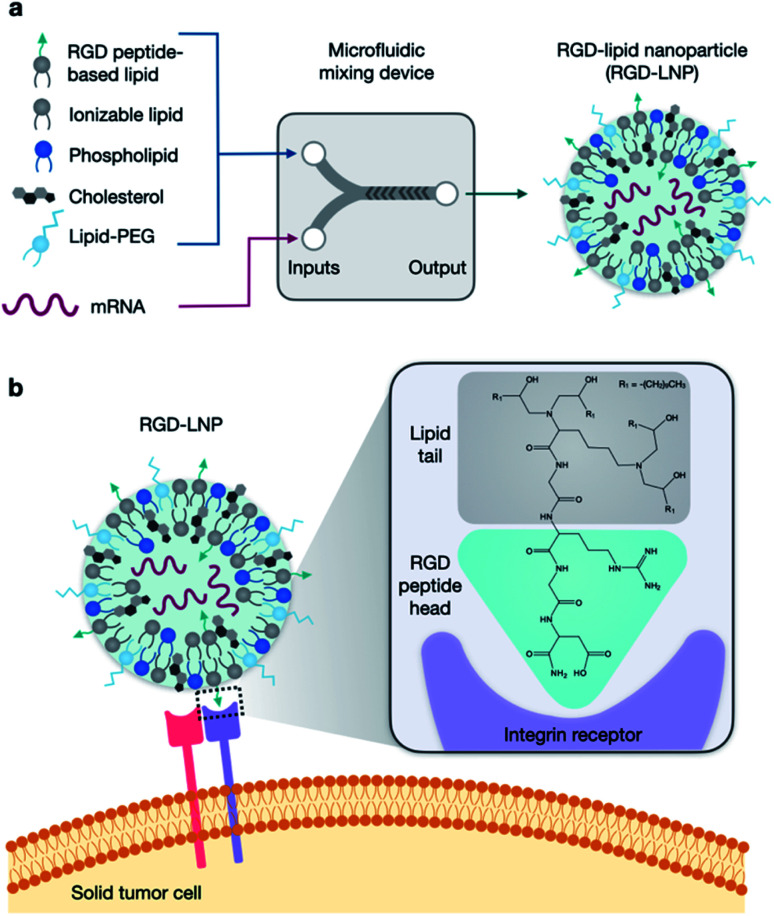
RGD-based lipid nanoparticles (RGD-LNP) for targeted mRNA delivery. (a) LNP components are prepared in two phases and combined *via* microfluidic mixing to form RGD-LNP. (b) RGD-LNP are expected to interact with integrin receptors on the surface of cancer cells.

Targeted delivery is of great interest in LNP studies due to its ability to enhance transfection, potentially overcoming current limitations of low genome editing efficiency and allowing for more specificity in delivery to organ and cell types of interest. Through targeting, LNPs can be developed to reach new cells and tissues, reduce toxicity and off target effects, and improve efficiency in difficult to transfect targets. In the literature, amino acids and peptides have been explored as a method to introduce targeting to the ionizable lipid component of LNPs.^29,30^ This method has shown successful, selective, and potent delivery of nucleic acid cargo.^31,32^ Here, we specifically examine Arg-Gly-Asp (RGD), the fibronectin tripeptide binding domain, as a potential peptide targeting moiety (Fig. 1). RGD is recognized by α_v_β_3_ and α_5_β_1_ integrins that can be overexpressed on several solid tumors, and integrins in general can mediate uptake into cells.^33^

In this work, we developed a library of 20 RGD-based ionizable lipids. These lipids, combined with DOPE, cholesterol, and lipid-PEG, were used to formulate 20 unique LNPs which were characterized and screened for luciferase mRNA delivery *in vitro*. Top performing RGD-lipids were mixed with C12-200 in various ratios to form 7 additional LNP formulations, which were screened similarly. The top LNP formulation, 1A RGD-based hybrid LNP, was found to effectively encapsulate and deliver mRNA *in vitro* and *in vivo*, with improved cell viability and whole-body delivery than the untargeted C12-200 control LNP. Further, this formulation was used to co-deliver Cas9 mRNA and sgRNA and was found to knockout green fluorescent protein (GFP) expression of HepG2 cells with efficiencies up to 90%. Overall, this RGD-lipid LNP formulation could provide a platform for protein replacement and gene editing applications with improved mRNA uptake.

## Materials and methods

### RGD peptide-based lipid synthesis

**Fig. 2:**
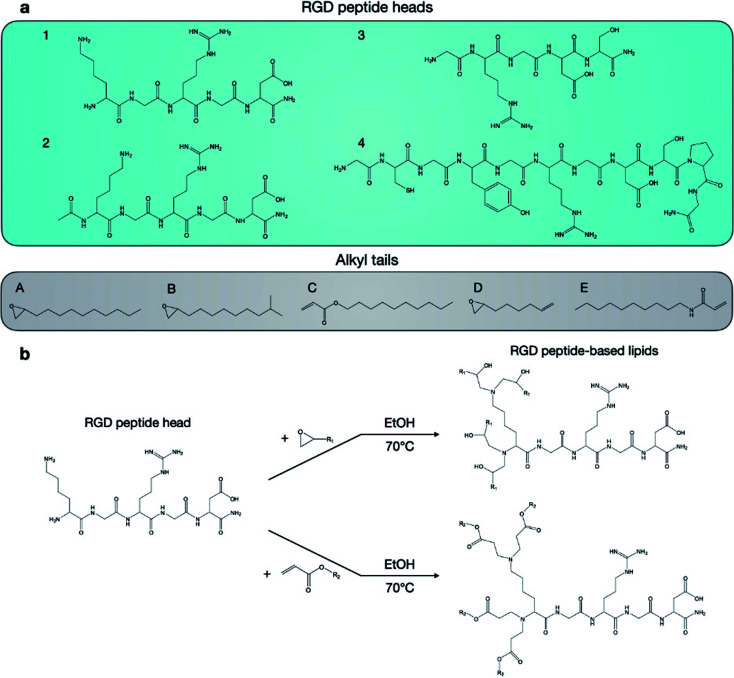
Synthesis of RGD peptide-based lipids by reaction between RGD peptide heads and alkyl tails. (A) Four RGD peptide heads and five alkyl tails were chosen for the formation of 20 unique RGD-peptide based lipids. (B) Lipids were synthesized by reacting heads and tails in ethanol for 3 days at 70 °C.

Twenty RGD peptide-based ionizable lipids as well as C12-200 were prepared *via* Michael addition or nucleophilic addition/SN_2_ reactions (Fig. 2). Note that the reaction between amine cores (1–5) with alkyl tails (C and E) in this library is regarded as a Michael addition reaction, while the reactions with epoxide (alkyl tails: A, B, and D) is considered a nucleophilic addition/SN_2_ reaction. Briefly, RGD peptides (GenScript, Piscataway, NJ) with reactive amino groups were dissolved in ethanol and combined with excess lipid tails (Sigma-Aldrich, St. Louis, MO) in a 4 mL glass vial under gentle stirring with a magnetic stir bar for 3 days at 70 °C. The reaction mixture was dried using a Rotovap R-300 (Buchi, New Castle, DE) and used for LNP formulation.

### mRNA synthesis

The firefly luciferase gene sequence was codon optimized, synthesized, and cloned into a proprietary mRNA production plasmid. Transcription was carried out using MegaScript T7 RNA polymerase (Invitrogen, Waltham, MA), and mRNA was precipitated using lithium chloride, purified by cellulose chromatography, and stored frozen at −80 °C for future use.

### LNP formulation

Ionizable lipid (RGD peptide-based lipid or C12-200) was combined in an ethanol phase with cholesterol (Sigma-Aldrich), 1,2-dioleoyl-*sn*-glycero-3-phosphoethanolamine (DOPE, Avanti, Alabaster, AL), and 1,2-dimyristoyl-*sn*-glycero-3-phosphoethanolamine-*N*-[methoxy(polyethylene glycol)-2000] (ammonium salt) (C14-PEG2000, Avanti) to a total volume of 100 μL. 25 μg of luciferase mRNA was prepared in 10 mM citrate buffer (pH = 3) to a total volume of 300 μL. A 10 : 1 weight ratio of ionizable lipid to luciferase mRNA was used to ensure encapsulation *via* electrostatic interactions. A syringe pump was used to combine the ethanol and citrate phases in a microfluidic device designed with herringbone features to formulate mRNA-containing LNPs *via* chaotic mixing.^34^ Then, LNPs were dialyzed against 1× PBS in cassettes with a molecular weight cutoff of 20 kDa for 2 h, filtered using a 0.22 μm filter, and stored at 4 °C for future use. All materials were prepared and handled ribonuclease-free throughout synthesis, formulation, and characterization steps. For gene editing experiments, Cas9 mRNA (TriLink BioTechnologies, San Diego, CA) and single-guide RNA (sgRNA) targeting EGFP (Axolabs, Kulmbach, Germany) were combined at ratios of 4 : 1 or 2 : 1 in 10 mM citrate buffer for LNP formulation.

### Dynamic light scattering

Dynamic light scattering (DLS) was used to evaluate LNP diameter and polydispersity using a ZetaSizer Nano Series (Nano ZS, Malvern Instruments, UK) at a scattering angle of 173°. 6 μL of each LNP solution was diluted 100× in 1× PBS in 4 mL disposable cuvettes (Malvern Panalytical, Malvern, UK). Three measurements each with at least 10 runs were recorded for each sample at room temperature.

### LNP p*K*_a_ measurements

Surface ionization measurements to calculate the p*K*_a_ of selected LNP formulations were performed as previously described.^35^ Briefly, buffered solutions containing 150 mM sodium chloride, 20 mM sodium phosphate, 20 mM ammonium acetate, and 25 mM ammonium citrate were prepared with pH measurements of 2 to 12 in increments of 0.5.5 μL of LNP and 125 μL of each pH-adjusted solution were added in black 96-well plates in triplicate. 2-(*p*-Toluidinyl)naphthalene-6-sulfonic acid (TNS) was then added to each well to a final TNS concentration of 6 μM. An Infinite 200 Pro plate reader (Tecan, Morrisville, NC) at an excitation wavelength of 322 nm and an emission wavelength of 431 nm was used to test the fluorescence intensity. Using least squares regression, the p*K*_a_ was taken as the pH corresponding to half-maximum fluorescence intensity, *i.e.*, 50% protonation.

### LNP encapsulation efficiency

mRNA encapsulation efficiency of each LNP formulation was calculated using the Quant-iTRiboGreen (Thermo Fisher Scientific, Waltham, MA) assay.^36^ LNP samples were diluted to approximately 2 ng μL^−1^ in microcentrifuge tubes containing either 1× TE buffer or 0.1% (v/v) Triton X-100 (Sigma-Aldrich). LNPs in TE buffer or Triton-X as well as mRNA standards were plated in triplicate in black 96-well plates following which fluorescent RiboGreen reagent was then added in each well. Fluorescence intensity was read on an Infinite 200 Pro plate reader (Tecan) at an excitation wavelength of 490 nm and an emission wavelength of 530 nm. Least squares linear regression (LSLR) was used to estimate a standard curve to quantify the RNA content. Encapsulation efficiency was calculated as the comparison of RNA content in TE buffer and in TX buffer.

### *In vitro* LNP-mediated luciferase mRNA delivery and cell viability in HepG2 cells

LNP-mediated *in vitro* transfection was evaluated in HepG2 cells seeded in 96-well plates at a density of 5000 cells per well in 100 μL of Dulbecco's Modified Eagle Medium (DMEM) media. Cells were seeded and cultured for 24 h at 37 °C before treatment in triplicate with a volume of LNPs corresponding to 10 ng of luciferase mRNA, including wells treated with C12-200 LNPs as a positive control. For the RGD binding studies, HepG2 cells were treated with excess RGD peptide (0.1 mg mL^−1^) for 1 h prior to the addition of RGD-lipid LNPs.^37^ 24 h following treatment with luciferase mRNA-containing LNPs, the medium was aspirated, cells were lysed with 20 μL per well of 1× lysis buffer (Promega, Madison, WI), and luciferase assay substrate (Promega) was added to each well. The luminescence intensity corresponding to luciferase protein expression was measured using a Infinite 200 Pro plate reader. Similarly, for cell viability experiments, 24 h following treatment with LNPs, cells were treated with CellTiter-Glo (Promega) and luminescence intensity corresponding to ATP production was measured using a Infinity 200 Pro plate reader. Luminescence measurements were normalized to untreated cells and a one-way ANOVA with the Dunnett correction for multiple comparisons was used to compare means between RGD LNP groups and C12-200 LNP.

### Luciferase imaging and quantification

To assess LNP biodistribution and *in vivo* transfection, eight-week old C57BL/6 mice were treated with either 0.1 mg kg^−1^ luciferase mRNA-containing RGD-lipid or C12-200 LNPs (*n* = 3) *via* tail vein injection. 6 h later, the mice received D-luciferin and potassium salt (Biotium, Fremont, CA) at 150 mg kg^−1^*via* intraperitoneal injection. 10 min later, mice were anesthetized using isoflurane and imaging was performed using an *in vivo* imaging system (IVIS, PerkinElmer, Waltham, MA) to evaluate whole-body luciferase expression. Next, mice were euthanized using CO_2_, organs were immediately collected, and bioluminescence imaging was performed using IVIS with 60 s exposure times.

Image analysis was conducted using the Living Image software (PerkinElmer). To quantify luminescence flux, a rectangular region of interest (ROI) was placed around the whole body or organ as well as in an area without any luminescence signal in the same image. Normalized flux was calculated by dividing the total flux from the whole body or organ ROI by the total flux from the background ROI. An unpaired, two-tailed *t* test was used to compare total flux between RGD and C12-200 only LNPs.

### *In vitro* gene editing in GFP^+^-HepG2 cells

GFP-expressing HepG2 cells were seeded in 6-well plates at a density of 100 000 cells per well and 24 h later, sgRNA/Cas9 mRNA-containing LNPs were used to treat cells. After 7 days, media was exchanged for fresh cell culture medium and GFP expression was evaluated using flow cytometry. GFP expression was normalized to untreated cells to determine gene editing efficiency and lipofectamine MessengerMAX (Thermo Fisher Scientific) was used as a positive control for the study. A one-way ANOVA with the Dunnett correction for multiple comparisons was used to compare means between the treatment groups and the GFP positive control.

### *In vivo* cytotoxicity

For cytotoxic evaluation of Luc-mRNA containing LNPs, plasma samples collected from mice 12 h after LNP or PBS injection were used to assess AST and ALT liver enzyme levels *via* colorimetric assay kits (Cayman Chemical, Ann Arbor, MI, USA). Mice AST/ALT data were normalized to the protein concentration in the sample as determined by the microBCA assay (Thermo Fisher, Waltham, MA).

### Animal studies

C57BL/6J mice were purchased from Jackson Laboratory. All animal procedures were performed in accordance with the Guidelines for Care and Use of Laboratory Animals of University of Pennsylvania and approved by the Animal Ethics Committee of University of Pennsylvania (protocol number 806540).

## Results and discussion

### Design, characterization, and *in vitro* evaluation of LNP library

In this report, we designed RGD peptide-based ionizable lipids for their potential to improve potency and targeting for LNP-mediated intracellular mRNA delivery (Fig. 1). A library of 20 RGD peptide-based ionizable lipids was synthesized by conjugating RGD peptide heads^1–4^ to alkyl lipid tails (A, B, C, D, and E) (Fig. 2).^38^ These RGD peptide-based ionizable lipids were then used to formulate LNPs and combined in an ethanol phase with three other excipients: (i) cholesterol, to enhance LNP stability and promote membrane fusion, (ii) DOPE, a helper lipid to fortify the bilayer structure of the LNP and promote endosomal escape, and (iii) lipid-anchored polyethylene glycol (C14-PEG2000), to reduce aggregation and nonspecific endocytosis (Fig. 1A).^39,40^ This ethanol phase was then combined with an aqueous phase containing mRNA using a microfluidic device to induce chaotic mixing and LNP formulation. The resulting LNP library was characterized using dynamic light scattering (DLS); the diameter of LNPs ranged from 50 to 200 nm and all LNPs had a polydispersity index (PDI) less than 0.3 (Table S1†). However, the mRNA encapsulation efficiency of these RGD peptide-based LNPs was lower than typically seen, with less than 50% encapsulation for all LNPs in the library.

**Fig. 3:**
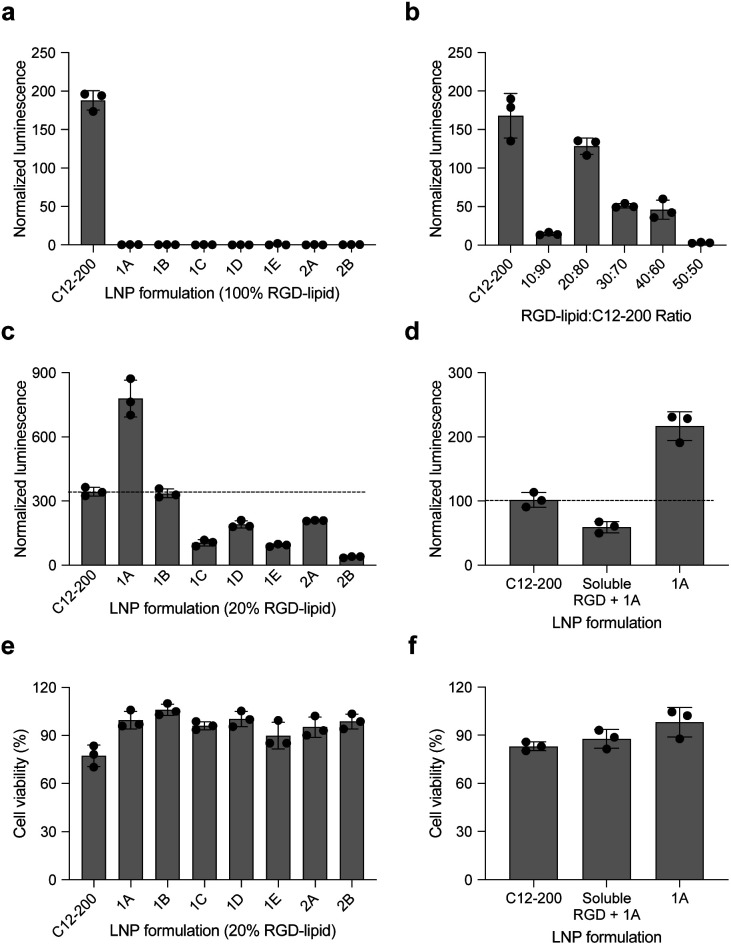
RGD-LNPs can be formulated to enhance mRNA transfection *in vitro* to HepG2 cells. (a) Luciferase expression of HepG2 cells treated with seven LNPs show little to no transfection when the ionizable lipid component is fully substituted with RGD peptide-based lipids. (b) Luciferase expression of HepG2 cells treated with LNPs containing C12-200 and the RGD peptide-based lipid 2A at various ratios. (c) Luciferase expression of HepG2 cells treated with seven different LNPs, incorporating RGD peptide-based lipids at the identified ratio of 20 : 80. (d) Luciferase expression of HepG2 cells treated with RGD-lipid LNPs (1A), soluble RGD + RGD-lipid LNPs (1A) and positive control sample C12-200 LNPs. (e) Cell viability of the seven different LNPs tested in (c). (f) Cell viability of the LNPs tested in (d). All results were normalized to untreated cells, three biological replicates for each sample. **p < 0.01, ***p < 0.001, ****p < 0.0001.

These LNPs were formulated with luciferase mRNA as a model cargo to evaluate *in vitro* mRNA delivery *via* bioluminescence. Upon intracellular uptake and endosomal escape, luciferase mRNA is translated into luciferase protein which reacts with luciferin reagent to produce bioluminescence as an indicator of functional mRNA delivery.^38^ HepG2 cells were selected to evaluate *in vitro* mRNA delivery as they are a commonly used immortalized cell line for drug metabolism and hepatotoxicity studies. Seven LNPs from the 20 LNP library – 1A, 1B, 1C, 1D, 1E, 2A, and 2B – were used to treat HepG2 cells and luciferase expression was evaluated 24 h later. The results demonstrate little to no transfection for the seven RGD peptide-based LNPs compared to C12-200 as a positive control, when the RGD peptide-based lipids are the only ionizable lipid in the LNP formulation (Fig. 3a).

Following these results, we formulated a small library of LNPs to evaluate the effect of partially substituting the C12-200 ionizable lipid with our 2A RGD peptide-based lipid at various RGD-lipid to C12-200 ratios (10 : 90, 20 : 80, 30 : 70, 40 : 60, and 50 : 50). Upon characterization of these LNPs, we found improved encapsulation efficiency for all formulations compared to their counterparts without C12-200 (Table S2†). Additionally, when screened *in vitro*, the 20 : 80 ratio of 2A RGD-lipid to C12-200 had more comparable delivery to C12-200 only LNPs than its counterpart LNP without C12-200 (Fig. 3b). As a result, we proceeded with a 20 : 80 ratio of RGD-lipid to C12-200 for further exploration due to its highest encapsulation efficiency and most comparable *in vitro* delivery to C12-200 only LNPs.

Therefore, we next formulated seven RGD peptide-based LNPs with a 20 : 80 ratio of RGD-lipid to C12-200. The characterization results demonstrate similar diameter and PDI measurements to C12-200 only LNPs with encapsulation efficiencies greater than 75% (Table S3†). For example, the diameter of LNPs formulated with RGD peptide-based lipid 1A had a diameter of 106.7 nm (Fig. S1†), a pK_a_ value of 6.41 (Fig. S2†), and an mRNA encapsulation efficiency of 89.49%, all of which are similar to our positive control C12-200 LNPs. These results confirm the successful formulation of LNPs with RGD peptide-based lipids with desirable physicochemical properties and efficient mRNA encapsulation for further investigation in different biological applications.

These seven RGD peptide-based LNPs – 1A, 1B, 1C, 1D, 1E, 2A, and 2B – formulated with a 20 : 80 ratio of RGD-lipid to C12-200, all showed some level of mRNA delivery and resulting luminescent signal. LNPs formulated with a 20% substitution of RGD peptide-based lipid 1A demonstrated significantly higher (****p < 0.0001) luminescence signal than the C12-200 only control (Fig. 3c). Ligand–receptor interactions play an important role in many biological processes. RGD containing peptides are known to mimic the binding domain of the extracellular matrix protein fibronectin and selectively bind to a subset of integrin receptors. Integrins are a class of transmembrane receptors expressed in various cell types that are involved in tumor progression.^33^ Here, we used HepG2 cancer cells to evaluate cell uptake. According to confocal images (Fig. S3†), the top performing RGD-based hybrid LNP 1A exhibited greater uptake by HepG2 cells compared to C12-200 LNPs, supporting the delivery results shown here. In terms of cell viability, six of the seven RGD LNPs had significantly improved (**p < 0.01, ***p < 0.001, ****p < 0.0001) cell viability compared to the C12-200 only control (Fig. 3e). Taken together, these results prompted us to select the C12-RGD (1A) LNP as our lead formulation due to its increased luciferase mRNA delivery and decreased toxicity compared to the C12-200 control.

Finally, to demonstrate whether the improved delivery efficacy with this RGD-LNP formulation is RGD-dependent, HepG2 cells were pre-treated with excess soluble RGD 1 h before the addition of 1A RGD-based hybrid LNPs. RGD will bind with the integrin receptor, which may block RGD-based lipid binding with integrins and the resulting RGD-LNP cell uptake. Again, the luciferase expression for our RGD LNPs without pre-treatment was significantly higher (***p < 0.001) than the C12-200 only control. Additionally, luciferase expression was reduced in the group pre-treated with the RGD peptide (Fig. 3d). There were no changes in cell viability upon pre-treatment with soluble RGD peptide (Fig. 3f). These results suggests integrin-RGD binding plays a key role in the intracellular uptake and luciferase expression of RGD-LNP.^37^

### *In vivo* evaluation of RGD peptide-based LNPs

**Fig. 4:**
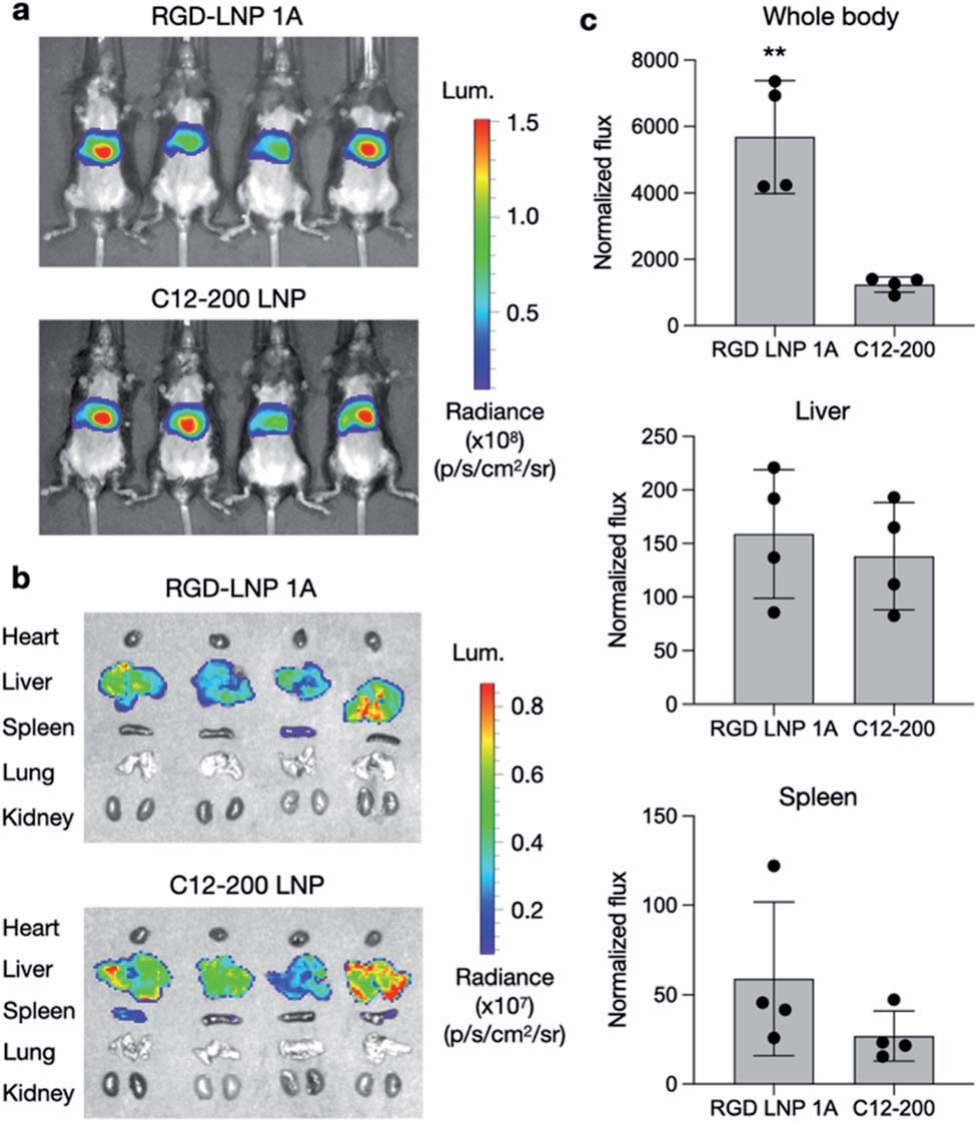
Luciferase mRNA delivery *in vivo*. (a) Whole body IVIS images of luciferase signal in mice after administration of LNPs. (b) Isolated organ IVIS images of luciferase signal, showing delivery to the liver and spleen. (c) Quantification of luciferase signal in the whole-body, liver, and spleen. Normalized total flux was averaged. *n* = 4 mice per treatment group. **p < 0.01.

To further assess the transfection efficiency of our novel RGD peptide-based LNPs *in vivo*, we evaluated LNP-mediated luciferase mRNA delivery for our lead 1A RGD lipid LNP compared to C12-200 only LNPs in mice. LNPs were administered to C57BL/6 mice *via* tail vein injection at a dose of 0.1 mg kg^−1^ of luciferase mRNA. Six hours after injection, D-luciferin was injected intraperitoneally and whole-body imaging was subsequently performed using an *in vivo* imaging system (IVIS) to quantify luciferase expression (Fig. 4a). Using ROI quantification of the whole-body images, there was significantly higher (**p < 0.01) luciferase expression between our RGD lipid LNPs and the C12-200 control (Fig. 4c). Next, the mice were euthanized and organs including the heart, liver, spleen, lung, and kidneys were isolated as evaluated for luciferase expression *via* IVIS (Fig. 4b). The images demonstrate LNP-mediated mRNA delivery to the liver and spleen in both groups. However, there was no significant difference in luciferase expression between the RGD lipid LNPs and C12-200 LNPs for either the liver or spleen (Fig. 4c).

Due to the high luciferase expression in the liver, we sought to explore LNP-mediated hepatotoxicity by quantifying the liver enzymes alanine aminotransferase (ALT) and aspartate aminotransferase (AST) in mouse plasma 12 h following LNP administration. There was no significant difference in ALT or AST levels for either the RGD lipid LNP or C12-200 LNP groups compared to the PBS control (Fig. S4†). This indicates that LNPs formulated with RGD peptide-based lipids enable efficient mRNA delivery to the liver without inducing enzyme release–associated toxicity. Overall, our RGD-lipid LNPs have low toxicity and higher cell viability compared to C12-200 LNPs.

### *In vitro* gene editing in GFP expressing HepG2 cells

**Fig. 5:**
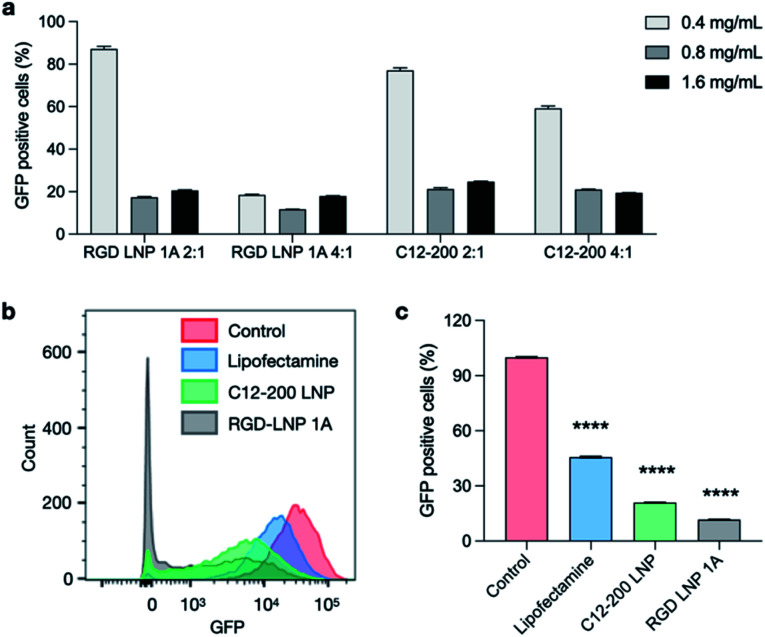
Co-delivery of Cas9 mRNA and sgRNA for *in vitro* gene editing in GFP^+^-HepG2 cells. RGD-lipid LNPs and C12-200 LNPs with various Cas9 mRNA/sgRNA ratios and LNP concentrations were selected. (a) GFP expression of HepG2 cells post treatment with LNPs co-delivering Cas9 mRNA and sgRNA. (b) GFP expression of HepG2 cells was assessed using flow cytometry. Cytometry plot shows decreased GFP expression in cells treated with 0.8 mg μL^−1^ RGD-lipid LNPs and C12-200 LNPs at Cas9 mRNA/sgRNA of 4 : 1. (c) Quantification of flow cytometry shows that cells treated with RGD-lipid LNPs have lower GFP expression, compared to untreated cells, lipofectamine control, and C12-200 LNPs. ****p < 0.0001.

To evaluate an additional biological application of our lead RGD peptide-based lipid, LNPs were formulated with either 20% of 1A RGD lipid or C12-200 alone to deliver Cas9 mRNA and sgRNA for *in vitro* gene editing in GFP expressing HepG2 cells. 24 h after seeding, cells were treated with LNPs at three different doses (0.4, 0.8, and 1.6 mg mL^−1^) and two different Cas9 mRNA to sgRNA ratios (4 : 1 and 2 : 1). Lipofectamine MessengerMAX was used as an industry standard transfection reagent to serve as a positive control. In typical mRNA delivery applications, LNPs only need to release mRNA into cytoplasm for its translation into functional proteins. However, since the functional protein Cas9, and the resulting Cas9/sgRNA complex targets DNA in the nucleus, gene editing applications require not only cytoplasm delivery, but trafficking to the nucleus. Further, changes to the genome do not immediately result in changes to the protein composition within the cell. In this work, editing cannot be measured using flow cytometry until a cycle of protein degradation and new protein translation has occurred. Therefore, we did not detect any obvious editing profiles at day one and day three. Seven days post-treatment, *in vitro* gene editing was assessed using flow cytometry (Fig. S5†). The results demonstrate that GFP knockout is dependent on total RNA dose (Fig. 5a) and that treatment with RGD-lipid LNPs reduces GFP mean fluorescence intensity (MFI) beyond the C12-200 and lipofectamine groups (Fig. 5b). Of note, RGD-lipid LNP at a dose of 0.8 mg mL^−1^ and a 4 : 1 ratio of Cas9 mRNA to sgRNA induced up to 90% knockout of GFP expression compared to the untreated control (Fig. 5c). Together, these results suggest that our RGD peptide-based lipid may expand the therapeutic potential of LNPs for mRNA therapeutics and CRISPR/Cas9 gene editing.

## Conclusions

The RGD peptide-based lipids introduced in this work are a potential novel approach in designing LNPs for targeted nucleic acid delivery. This multicomponent delivery system shows efficient mRNA encapsulation, significant transfection efficiency of nucleic acids, and an excellent safety profile. The introduction of this RGD-peptide lipid into a C12-200 LNP formulation demonstrated improved cellular uptake and lower toxicity when compared to LNPs formulated with C12-200 alone. Additionally, RGD lipid LNPs were selected to co-deliver Cas9 mRNA and sgRNA for *in vitro* gene editing, successfully knocking out green fluorescent protein (GFP) expression in up to 90% of HepG2 cells. This RGD-LNP system shows significant potential both *in vitro* and *in vivo*, not only improving RNA delivery efficacy, but also potentially opening a new avenue for targeted delivery of other nucleic acids into specific cells and tissues of interest. This technology may be applicable for potential *in vivo* gene editing applications or for mRNA therapeutic delivery in tumor-bearing mouse models.

## Author contributions

J. Q. and L. X. were responsible for conceptualization of this work and initial manuscript writing. J. Q., L. X., N. G., and H. Z. were responsible for data collection and initial analysis. J. Q, S. J. S., and K. L. S. were responsible for data analysis and the production of graphs. S. J. S., R. M. H., and K. L. S. were responsible for manuscript writing, review, and editing.

## Conflicts of interest

There are no conflicts to declare.

## Supplementary Material

RA-012-D2RA02771B-s001
