# Supplementary material for: RGD peptide-based lipids for targeted mRNA delivery and gene editing applications
Source: RSC Adv. 2022 Sep 7;12(39):25397–404. doi: 10.1039/d2ra02771b (PMC9450108; doi:10.1039/d2ra02771b)
Supplement: RA-012-D2RA02771B-s001 [file RA-012-D2RA02771B-s001.pdf]

Electronic Supplementary Material (ESI) for RSC Advances.
This journal is © The Royal Society of Chemistry 2022

Electronic Supplementary Information (ESI)

RGD Peptide-based Lipids for Targeted mRNA Delivery and Gene Editing Application

Qin, et al.

Table S1. Physical characterization of RGD-LNPs

LNP formulation	Diameter (nm)	Polydispersity index (PDI)	Encapsulation efficiency (%)
1A	63.34	0.238	36.03
1B	75.73	0.036	21.29
1C	57.27	0.189	8.14
1D	77.09	0.043	41.78
1E	86.61	0.171	42.14
2A	82.83	0.270	48.15
2B	51.85	0.270	19.38
2C	64.09	0.078	6.34
2D	68.45	0.130	2.69
2E	224.1	0.109	1.57
3A	52.09	0.176	3.89
3B	46.68	0.174	6.93
3C	82.25	0.165	3.87
3D	109.6	0.219	6.50
3E	100.9	0.165	2.49
4A	95.46	0.297	3.78
4B	75.25	0.057	2.56
4C	58.43	0.088	2.47
4D	108.8	0.574	4.29
4E	93.68	0.076	5.32

Table S2. Physical characterization of RGD-LNPs with varying amounts of C12-200

LNP formulation	Diameter (nm)	Polydispersity index (PDI)	Encapsulation efficiency (%)
C12-200	79.09	0.152	89.11
1A	106.70	0.122	89.49
1B	88.77	0.061	89.62
1C	103.9	0.931	86.46
1D	92.54	0.088	81.14
1E	86.17	0.192	88.44
2A	130.70	0.224	75.06
2B	76.65	0.011	72.61

Table S3. Physical characterization of RGD-LNPs with the addition of C12-200

LNP	Diameter (nm)	Polydispersity index (PDI)	Encapsulation efficiency (%)
10% RGD-lipid + 90% C12-200	123.14	0.134	73.29
20% RGD-lipid + 80% C12-200	133.65	0.227	82.48
30% RGD-lipid + 70% C12-200	104.83	0.128	79.32
40% RGD-lipid + 60% C12-200	122.34	0.313	70.45
50% RGD-lipid + 50% C12-200	119.97	0.236	51.99

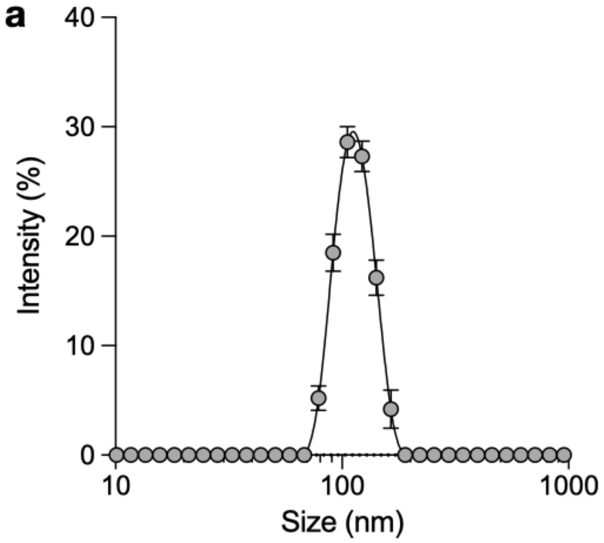

Figure S1. Dynamic light scattering (DLS) analysis of RGD-LNP 1A, showing the size and size distribution of n+3 measurements

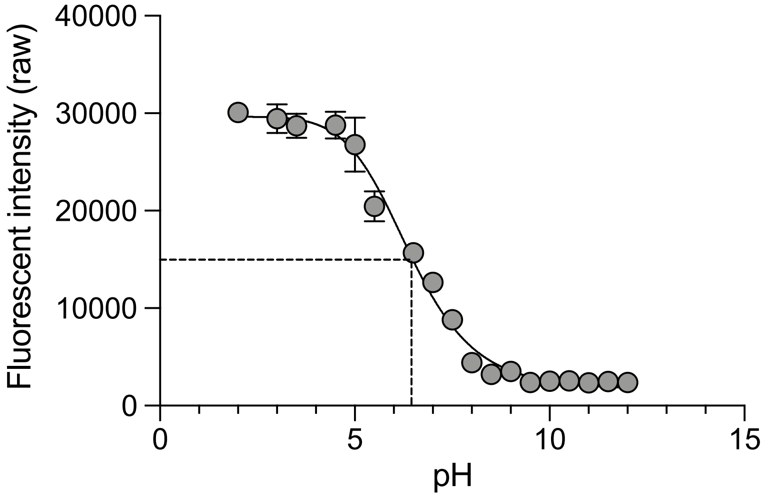

Figure S2. Result to determine RGD-lipid LNP (1A) pKa for the LNPs encapsulating luciferase mRNA. pKa is calculated as the pH corresponding to half of the maximum fluorescence value.

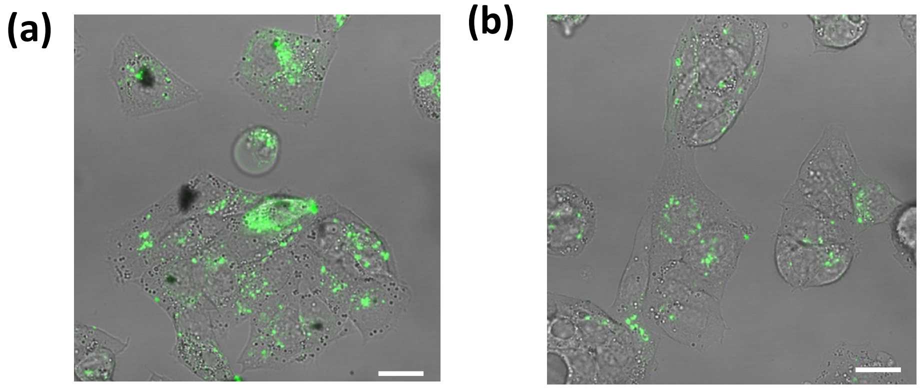

Figure S3. Confocal images (6h treat time with 1% DiO-labelling) to test the uptake of HepG2 cells for (A) C12-RGD hybrid LNP (lead LNP); (B) C12-200 LNP as a control. Scale bar: 20 μm.

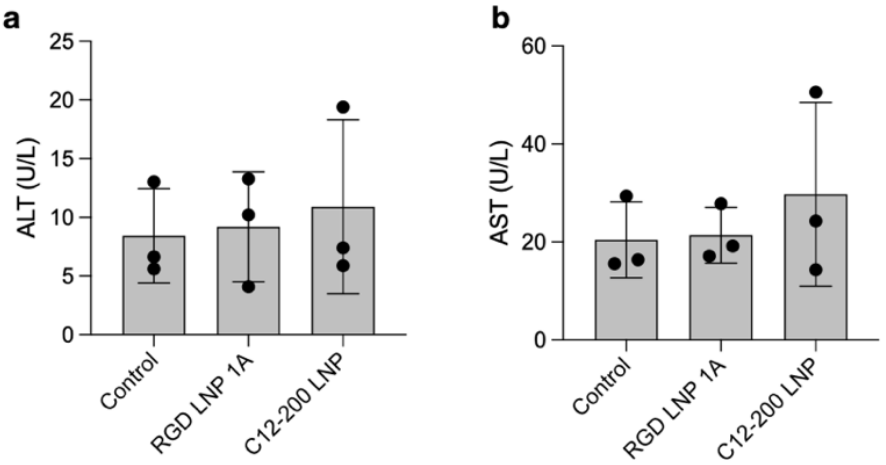

Figure S4. Liver enzymes for in vivo experiments indicates minimal toxicity

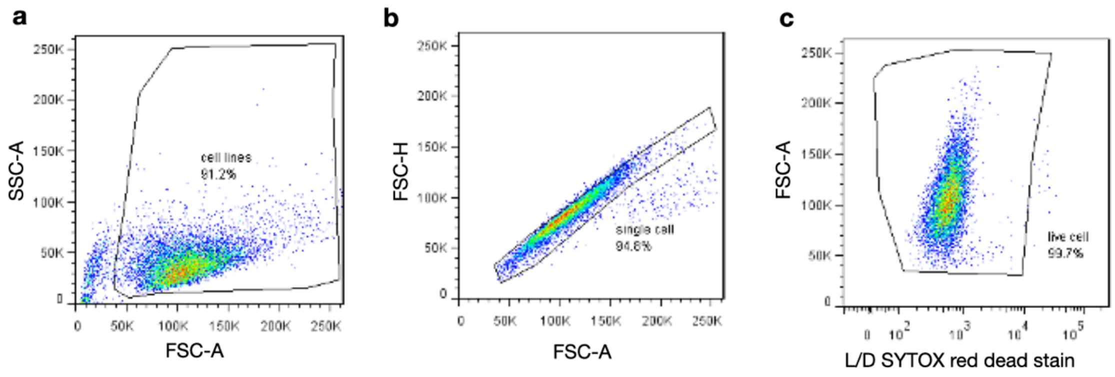

Figure S5. Flow gating strategy for knockdown of GFP expression in GFP+ HepG2 cells
